# Association of the Academic Performance of Undergraduate Medical Students With Positive Well-Being, Intelligence, and Factors of Academic Success

**DOI:** 10.7759/cureus.50077

**Published:** 2023-12-06

**Authors:** Sajida Agha, Abdullah Abdulrahman Alzayed, Tameem Ahmed Alfuraih, Fahad Turki Alenazi, Moath Ibrahim Alomair, Emad Masuadi

**Affiliations:** 1 Medical Education, King Saud Bin Abdulaziz University for Health Sciences, Riyadh, SAU; 2 General Practice, Eradah Mental Health Complex, Al-Baha, SAU; 3 Psychology, King Saud Bin Abdulaziz University for Health Sciences College of Medicine, Riyadh, SAU; 4 Biostatistics, King Saud Bin Abdulaziz University for Health Sciences, Riyadh, SAU

**Keywords:** well-being, medical students, intelligence, academic success, academic performance

## Abstract

Background: The academic performance of medical students may affect their competence in future career prospects. Developing students' subjective well-being to improve academic performance is complex and has been investigated for many years. This cross-sectional study assessed the relationship between academic performance, general intelligence, and medical students' positive well-being.

Methods: This cross-sectional, internet-based survey included male and female medical students from pre-clinical and clinical years at one of the largest public-sector universities in Riyadh, Saudi Arabia, between February 2020 and April 2020. The questionnaire included the World Health Organization-Five (WHO-5) Well-being Index, the Draw-A-Person Intellectual Ability (DAP: IQ) Test, and the Academic Success Inventory for College Students (ASICS). Academic performance was represented by a self-reported high-grade point average (GPA). Logistic regression was used to assess the association between academic performance and each of the well-being scores, intelligence, and academic success factors. Statistical significance was established at a p-value less than 0.05.

Results: Responses were received from 176 medical students. Most participants were males (93.8%). There was a significant association between GPA above 4.5 and the total WHO-5 well-being score (p = 0.013), the ASICS general skills (p = 0.007), perceiving instructor efficacy (p = 0.005), confidence (p < 0.001), personal adjustment (p = 0.023), and lack of anxiety (p = 0.006). No association was found between GPA and intelligence quotient (IQ) or well-being when other factors were adjusted.

Conclusions: Good academic performance is associated with subjective well-being and domains of academic success, such as perceived efficacy of the instructor, confidence, and personal adjustment. Implementing student development programs in medical schools can have a positive impact on students' academic performance and skills. Future studies assessing the different student support and development programs and their impact on academic success are needed.

## Introduction

The academic performance of university students represents a major concern for the students, the university administrators, and stakeholders. Students with good academic performance possess a higher ability to decide on their future careers and achieve success [1]. However, several student-related and instructor-related factors can impact the student’s academic achievement [2].

University students, particularly in medical colleges, face several challenges during their education. Students may suffer academic stress due to multiple exams, tutorials, and strict rules demanding adherence to professional behavior. Excessive stress can negatively impact medical students' psychological well-being [3], which is defined as “the presence of positive feelings (e.g., good self-esteem) or the absence of negative feelings (e.g., symptoms of depression or anxiety)” [4]. The model of Ryff and Singer [5] identified six dimensions for well-being, including autonomy, environmental mastery, personal growth, positive relations with others, purpose in life, and self-acceptance. Subjective well-being is closely related to quality of life (QoL) [6,7], and it is closely related to students’ academic performance [3].

Another factor impacting academic achievement is the student’s primary mental ability, which is usually represented by the intelligence quotient (IQ). However, the extent to which IQ impacts academic achievement is controversial [8,9].

Several other factors - known in some models as factors of academic success - have the potential to affect academic performance. These include internal and external motivation, study and exam habits, persistence, quantity of work, quality of study, learning styles, and adaptation to personal problems [10,11].

Few studies have assessed the association of academic achievement with well-being, mental ability, and academic success in medical students. The identification of factors affecting academic performance will help design specific programs to improve the students' performance. Therefore, the objective of this study was to assess the relationship between academic performance, general intelligence, and medical students' positive well-being at King Saud bin Abdulaziz University for Health Sciences.

## Materials and methods

Ethical considerations

The institutional review board approved the study (IRB # SP18\282\R). Participation in the study was voluntary, and informed consent was acquired from each participant before the administration of the questionnaire. The data collection sheet was kept anonymous to preserve the confidentiality of the participants, and they were assured that their data and responses would be used only in the current study and not disclosed to any third party.

Study design, date, and setting

This cross-sectional, survey-based study enrolled male and female medical students from pre-clinical and clinical years at one of the largest public-sector universities in Riyadh, Saudi Arabia. It was conducted between February 2020 and April 2020. The college offers undergraduate and postgraduate programs. The campus has two separate branches for male and female students. Integration of the basic sciences with clinical medicine is the keynote of the educational program. The curriculum, collectively overseen by the faculty in basic sciences and clinical domains, is delivered to small student groups through a problem-based learning approach.

Eligibility criteria

The study included male and female undergraduate students from both pre-clinical and clinical years at a public-sector medical college who consented to participate in the study and completed the study questionnaire. Graduates and students of postgraduate medical studies were excluded.

Study tools

Data were collected using a questionnaire, which was divided into three parts to assess the association between well-being, academic success, and intelligence.

Part 1 consisted of demographic information, including age, gender, marital status, level of study, and grade point average (GPA). The academic performance of medical students was measured by their GPA, which is one of the most common indicators of academic performance [12]. For this study, grades were self-reported as the overall GPA record maintained by the assessment unit was not accessible due to the confidentiality policy of the respective medical college. The students’ GPAs were categorized into two levels (≤4.5 and >4.5).

Part 2 consisted of two self-administered tools that were used to assess the level of well-being and academic success. Psychological well-being was assessed using the World Health Organization-Five Well-being Index (WHO-5), which consists of five items on a six-point Likert scale. The WHO-5 was developed with the support of the World Health Organization Collaborating Centre at the Psychiatric Research Unit, Mental Health Centre North Zealand, Hillerød, Denmark. The total score ranges between 0 and 100, where obtaining a score of <50 is interpreted as poor well-being, and the higher score means better well-being [13,14]. We used the Arabic version of WHO-5, which was previously developed and validated and had a Cronbach’s alpha of 0.91, indicating good internal consistency [15]. The factors imperative for students' academic success were assessed using the Academic Success Inventory for College Students (ASICS). The ASICS includes 50 items that encompass nine factors: general academic skills, career decision-making, internal and external motivation, lack of anxiety, concentration, socializing, personal adjustment, and perceived efficacy of the instructor. Cronbach’s alpha values for the nine factors of ASICS range from 0.62 to 0.93 [10,16]. In the present study, we included 22 items from ASICS with high predictive validity [10,17]. Permission to use the questionnaires for the present study was received from the authors through email, as the questionnaires are not available online for use.

In the third part, general intelligence was assessed using the Draw-A-Person Intellectual Ability (DAP: IQ) Test by Reynolds et al. to estimate adults' cognitive and intellectual ability. The DAP test was reported to have a Cronbach’s alpha of 0.82 for the 23 items [18,19].

A pilot study was conducted on 30 students to assess the reliability of the two scales in our sample. The overall Cronbach’s alpha for both scales was 0.87. Results from the pilot study were used to enhance the clarity of the questions and were not added to the final data analysis.

The questionnaires, with guidelines and informed consent forms, were sent electronically through an institutional email circular, followed by three email reminders. Participation was voluntary, and no incentives were offered. The whole process of completing the questionnaire was estimated to take 15-20 minutes.

Sample size

All male and female medical students (N = 1200) were called to participate in the study. The sample size was calculated using Roasoft software [20]. The required sample size was 377 with a 95% confidence level and a 5% margin of error based on an expected response level of 50%.

Statistical analysis

Statistical analysis was performed using the Statistical Package for the Social Sciences (SPSS) at IBM version 22 (IBM Corp., Armonk, NY) [21]. Categorical data (such as gender, level of study, and entry-level) were presented as counts and percentages, and their association with GPA groups was assessed using Pearson’s Chi-square test, Fisher’s exact test (for nominal variables), or Cochran Armitage test for trend (for ordinal variables). Numerical data were summarized as the median and standard deviation (SD), and the difference between the GPA groups was tested using the independent samples T-test. Univariate binomial logistic regression analysis was conducted with the outcome of GPA>4.5 and the independent variables including all students’ demographics as well as the IQ, WHO-5 wellbeing score, and ASICS scores. Spearman’s rank-order correlation was done to assess the relationship between well-being and the domains of ASICS. A p-value of ≤0.05 was considered significant for all the statistical tests.

## Results

Among the 377 participants invited, 176 students completed the surveys, resulting in a response rate of 46.7%. The age of most students ranged between 20 and 25 years (n = 164, 93.2%), whereas each of the age groups "15-19 years" and "26-30 years" were six in number (3.4%). Among the respondents, 165 (93.8%) were males and 172 (97.7%) were single. Medical students from pre-clinical years were 122 (69.3%). Most students were admitted to the college as graduates of high school (91.5%). Eighty-eight students (50%) had a GPA above 4.5, while 64 (36.4%) had a GPA ranging between 4.01 and 4.5, and 24 students had a GPA of less than 4. The socio-demographic characteristics of the study subjects are detailed in Table 1.

**Table 1:** Participants’ characteristics (N = 176). N: Total number of participants.

Respondents’ characteristics		N	%
Age	15–19 years	6	3.4
	20–25 years	164	93.2
	26–30 years	6	3.4
Gender	Male	165	93.8
	Female	11	6.3
Marital status	Single	172	97.7
	Married	4	2.3
Year in medical school	1^st^-year pre-clinical	71	40.3
	2^nd^-year pre-clinical	51	29.0
	1^st^-year clinical	21	11.9
	2^nd^-year clinical	33	18.8
Streams	High school graduate	161	91.5
	Bachelor	15	8.5
GPA	<4.0	24	13.6
	4.01–4.5	64	36.4
	>4.5	88	50.0

**Figure 1:**
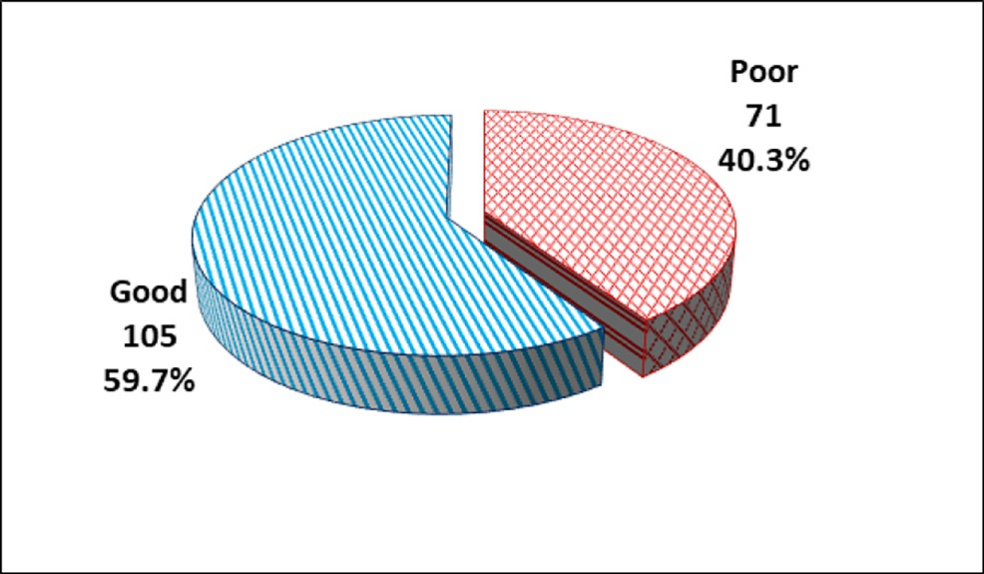
Subjective well-being in the enrolled students (N = 176).

According to the total WHO-5 well-being score, 105 participants (59.7%) had good subjective well-being (score >50), while 71 participants (40.3%) had poor well-being (score <50; Figure 1).

When the respondents were categorized into two groups (GPA ≤ 4.5 and GPA > 4.5), we found that no significant differences existed regarding age, gender, marital status, year of study, or stream between the groups (all p-values > 0.5; Table 2).

**Table 2:** Comparison between low and high GPA groups (N = 176). N: total number of participants; ^#^Cochran-Armitage test for trend; ^##^Pearson’s Chi-square test for independence of observations/Fisher’s exact test.

Respondents’ characteristics		GPA ≤ 4.5 N = 88	GPA > 4.5 N = 88	p-value
Age	15–19	3 (3.4%)	3 (3.4%)	0.565^#^
	20–25	81 (92.0%)	83 (94.3%)	
	26–30	4 (4.5%)	2 (2.3%)	
Gender	Male	80 (90.9%)	85 (96.6%)	0.119^##^
	Female	8 (9.1%)	3 (3.4%)	
Marital status	Single	86 (97.7%)	86 (97.7%)	1.000^#^
	Married	2 (2.3%)	2 (2.3%)	
Year of study	1^st^-year pre-clinical	35 (39.8%)	36 (40.9%)	0.181^#^
	2^nd^-year pre-clinical	32 (36.4%)	19 (21.6%)	
	1^st^-year clinical	9 (10.2%)	12 (13.6%)	
	2^nd^-year clinical	12 (13.6%)	21 (23.9%)	
Stream	High school graduate	77 (87.5%)	84 (95.5%)	0.059^#^
	Bachelor	11 (12.5%)	4 (4.5%)	

**Figure 2:**
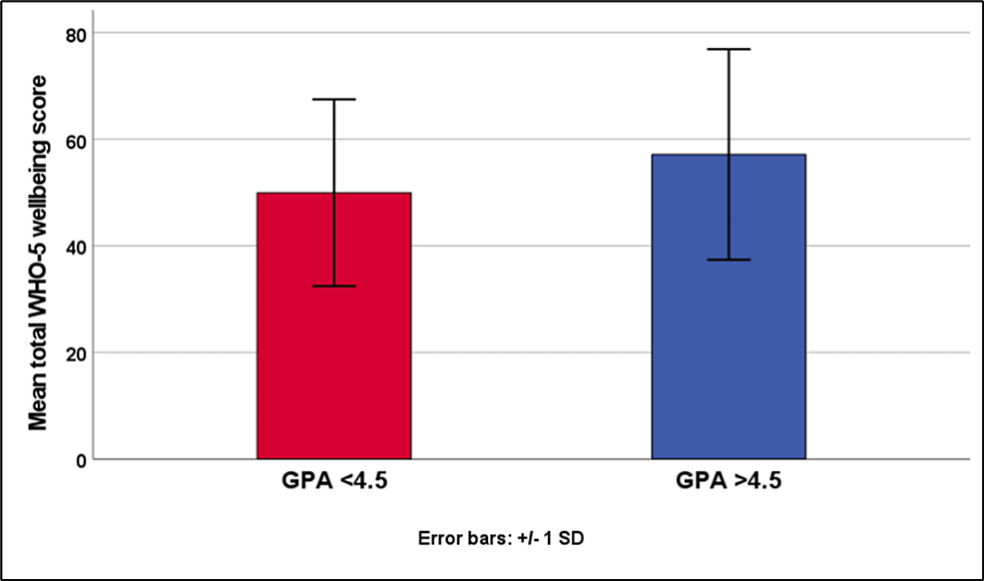
Comparison of mean total WHO-5 Well-Being Score between the GPA groups. Error bars represent ± 1 standard deviation.

The comparison of IQ showed no significant difference between the two groups (p = 0.584). The GPA > 4.5 group showed a significantly higher means of the total WHO-5 score (p = 0.012) as well as the ASICS components of skills (p = 0.006), perceiving instructor efficacy (p = 0.004), confidence (p < 0.001), personal adjustment (p = 0.021), and lack of anxiety (p = 0.005; Table 3, Figure 2).

**Table 3:** Comparison of intelligence, domains of academic success, and well-being between low and high GPA groups (n = 176). N: total number of participants; ^#^independent samples T-test; ^##^Pearson’s Chi-square test for independence of observations; *significant at p≤0.05.

Studied scores		GPA ≤ 4.5 N = 88	GPA > 4.5 N = 88	p-value #
IQ	Mean ± SD	111.6 ± 17.2	113.1 ± 18.7	0.584^#^
	Min–Max	86.0–147.0	89.0–147.0	
Total WHO-5	Mean ± SD	50.0 ± 17.5	57.1 ± 19.7	0.012*^#^
	Min–Max	4.0–88.0	12.0–100.0	
	Poor	42 (47.7%)	29 (33.0%)	0.046*^##^
	Good	46 (52.3%)	59 (67.0%)	
ASICS Skills	Mean ± SD	6.9 ± 2.5	8.0 ± 3.1	0.006*^#^
	Min–Max	2.0–12.0	2.0–14.0	
ASICS Instructor	Mean ± SD	9.9 ± 3.3	11.5 ± 4.1	0.004*^#^
	Min–Max	3.0–18.0	3.0–21.0	
ASICS Career Decidedness	Mean ± SD	8.3 ± 3.4	8.3 ± 3.8	0.917^#^
	Min–Max	2.0–14.0	2.0–14.0	
ASICS External Motivation	Mean ± SD	16.2 ± 3.0	16.2 ± 3.5	0.944^#^
	Min–Max	5.0–21.0	3.0–21.0	
ASICS Confidence	Mean ± SD	12.9 ± 3.9	15.5 ± 3.4	<0.001*^#^
	Min–Max	4.0–21.0	3.0–21.0	
ASICS Personal Adjustment	Mean ± SD	6.4 ± 2.9	7.4 ± 2.9	0.021*^#^
	Min–Max	2.0–14.0	2.0–14.0	
ASICS Concentration	Mean ± SD	8.2 ± 1.8	8.5 ± 2.3	0.326^#^
	Min–Max	2.0–13.0	2.0–14.0	
ASICS Socializing	Mean ± SD	8.3 ± 3.2	8.6 ± 3.2	0.605^#^
	Min–Max	2.0–14.0	2.0–14.0	
ASICS Internal Motivation	Mean ± SD	5.2 ± 1.5	5.4 ± 1.5	0.254^#^
	Min–Max	1.0–7.0	1.0–7.0	
ASICS Lack of Anxiety	Mean ± SD	6.2 ± 2.5	7.4 ± 3.0	0.005*^#^
	Min–Max	2.0–12.0	2.0–14.0	

The results of univariate logistic regression analysis showed that the likelihood of having a GPA > 4.5 was significantly increased with higher scores of the total WHO-5 wellbeing score (cOR: 1.021, 95% CI: 1.004-1.038, p = 0.013), the ASICS skill (cOR: 1.161, 95% CI: 1.041-1.295, p = 0.007), instructor (cOR: 1.128, 95% CI: 1.038-1.226, p = 0.005), confidence (cOR: 1.213, 95% CI: 1.109-1.328, p < 0.001), personal adjustment (cOR: 1.129, 95% CI: 1.017-1.252, p = 0.023), and lack of anxiety (cOR: 1.166, 95% CI: 1.044-1.303, p = 0.006; Table 4).

**Table 4:** Univariate logistic regression analysis of factors potentially contributing to high GPA (N = 176). CI: confidence interval; cOR: crude odds ratio; *significant at p≤0.05.

Independent variables		Reference category	p-value	cOR	95% CI of OR
Age	20–25	15–19	0.977	1.025	0.201–5.226
	26–30		0.560	0.500	0.049–5.154
Gender	Male	Female	0.134	2.833	0.726–11.057
Year of study	2^nd^ year preclinical	1^st^ year preclinical	0.142	0.577	0.277–1.203
	1^st^ year clinical		0.604	1.296	0.486–3.459
	2^nd^ year clinical		0.219	1.701	0.728–3.974
Stream	Bachelor	High school graduate	0.069	0.333	0.102–1.091
Marital status	Married	Single	1.000	1.000	0.138–7.262
Total WHO-5	Numerical	-	0.013*	1.021	1.004–1.0381
IQ	Numerical	-	0.581	1.005	0.988–1.021
ASICS Skills	Numerical	-	0.007*	1.161	1.041–1.2951
ASICS Instructor	Numerical	-	0.005*	1.128	1.038–1.226
ASICS Career Decidedness	Numerical	-	0.916	0.996	0.917–1.081
ASICS External Motivation	Numerical	-	0.944	0.997	0.909–1.093
ASICS Confidence	Numerical	-	<0.001*	1.213	1.109–1.328
ASICS Personal Adjustment	Numerical	-	0.023*	1.129	1.017–1.252
ASICS Concentration	Numerical	-	0.325	1.076	0.930–1.244
ASICS Socializing	Numerical	-	0.603	1.025	0.934–1.125
ASICS Internal Motivation	Numerical	-	0.253	1.122	0.921–1.368
ASICS Lack of Anxiety	Numerical	-	0.006*	1.166	1.044–1.303

The total WHO-5 wellbeing score showed a significant positive, though weak, correlation with the ASICS components of skill (rs=0.246, p=0.001), career decidedness (rs=0.267, p<0.001), confidence (rs=0.268, p<0.001), personal adjustment (rs=0.230, p=0.002), and lack of anxiety (rs=0.206, p=0.006; Table 5).

**Table 5:** Correlation between well-being and the domains of the academic success inventory for college students. rs: Spearman’s rank-order correlation; *significant at p≤0.05.

	Total WHO-5 well-being score	
	r_s_	p-value
ASICS: Skills	0.246	0.001*
ASICS: Instructor	0.013	0.869
ASICS: Career Decidedness	0.267	<0.001*
ASICS: External Motivation	−0.036	0.636
ASICS: Confidence	0.268	<0.001*
ASICS: Personal Adjustment	0.230	0.002*
ASICS: Concentration	0.145	0.054
ASICS: Socializing	−0.035	0.642
ASICS: Internal Motivation	0.007	0.928
ASICS: Lack of Anxiety	0.206	0.006*
IQ	0.100	0.185

## Discussion

The present study assessed the association of subjective well-being, general intelligence, and academic success with the academic performance of medical students.

The present study found no significant association of IQ with academic performance (p = 0.584), which agrees with earlier reports [22-24]. On the contrary, other studies suggested a positive influence of IQ on the students’ academic achievement [25,26]. The controversial relationship between IQ and academic performance may be attributed to differences in the population from which the participants were sampled. Various studies included students from different faculties and specialties. One study found that IQ correlated with GPA in the total sample but not in medicine, information technology, or software engineering students [22]. It seems that general intelligence would not significantly affect individuals' performance abilities that motivate them to succeed in life.

The current study found no significant association between gender and academic performance (p = 0.134); however, most respondents were male students, and this may mask gender differences that exist. We also found that the GPA was neither significantly associated with the academic year of the students (p = 0.181) nor with the stream from which the students were admitted into the college (p = 0.059). In agreement with these findings, an earlier study in Saudi Arabia [11] found no significant association between gender, academic year, and academic performance. However, Alzahrani et al. [27] reported a significant association between GPA and the academic year of the student, where students in the second and third years tended to have a low GPA. These differences could be attributed to the use of different study tools to assess academic performance. Also, differences in the distributions of educational curricula, academic assignments, and exams may affect the association of academic performance with academic years.

In the current study, a significantly higher percentage of students with high GPAs had good well-being compared to those with low GPAs (67% vs. 52.3%, p = 0.046). This finding agrees with several earlier studies, which identified a weak positive relationship between well-being and academic performance [28-30]. Moreover, Shareef et al. [31] reported a direct relationship between the academic performance of medical students and their QoL. According to the results of the current study, increasing the score of subjective well-being by ten points increases the chance of getting a high GPA by 21%. Potential research should focus on identifying and comparing the methods that enhance the well-being of medical students so that they can improve their academic performance.

The relationship between GPA and academic success is an area of contradiction among researchers. We found a significant association between five components of the ASICS tool and GPA. These included the domains of general academic skills, perceiving instructor efficacy, confidence, personal adjustment, and lack of anxiety. Similarly, Wigtil and Henriques [32] reported an association between academic success and performance. Thus, students with a high GPA tend to display prominent internal and external motivation and focus on achieving academic goals. They trust their tutors' competence, display less anxiety, and adjust easily to the extreme demands of the medical program. Comprehending the factors related to academic success could offer valuable insights for shaping and executing initiatives to enhance the professional and healthcare skills of medical students.

Academic success factors seem to be related to subjective well-being. The present study revealed a positive relationship between well-being and other components of academic success, including general skills, career decision-making, confidence, personal adjustment, and lack of anxiety. Likewise, a study conducted in China reported a negative effect of anxiety on well-being [33]. In addition, personal life events exhibited a solid relationship to professional burnout, affecting positive well-being [34].

We chose not to perform multivariate logistic regression analysis, partly because of the non-significant association of baseline characteristics (age, gender, academic year) with GPA and partly because of the perceived correlation between well-being and some of the ASICS domains. Thus, entering the well-being score and the ASICS domain scores into a multivariate model may negate each other's effect on GPA.

To the best of our knowledge, this study is the first to shed light on general intelligence, subjective well-being, academic success, and academic performance in undergraduate medical students in Saudi Arabia. Some limitations of the study need to be highlighted. Although our sample strategy focused on the maximum number of students to collect a potentially wide range of responses, the response rate was only 46.7% due to the COVID-19 situation. The low response rate and non-response bias might affect this study’s findings. Also, the GPA was self-reported, and the absence of objective evidence may introduce potential bias in the current study. Furthermore, our study was conducted at a single center, which may limit the generalizability of its results, and its cross-sectional design does not allow for establishing the potential cause-effect relationships between the study variables. Hence, these limitations should be taken into consideration when interpreting the results of the present study.

## Conclusions

Good academic performance is significantly associated with subjective well-being and domains of academic success, such as the perceived efficacy of the instructor, confidence, and personal adjustment. Implementing student development programs in medical schools can positively impact students' academic performance and skills. Educational institutions are recommended to prioritize both academic success and well-being domains, recognizing their significant impact on their students' academic accomplishments. A greater focus should be placed on cultivating soft skills such as empathy, interpersonal skills, personal adjustment, and motivation. Future multicenter research is recommended in this less-explored area before drawing definitive conclusions that can be broadly applied. Further research with a larger and more diverse sample and a longitudinal design may help strengthen the conclusions.
